# Effect of whole-hand water flow stimulation on the neural balance between excitation and inhibition in the primary somatosensory cortex

**DOI:** 10.3389/fnhum.2022.962936

**Published:** 2022-10-25

**Authors:** Dat Le Cong, Daisuke Sato, Koyuki Ikarashi, Tomomi Fujimoto, Genta Ochi, Koya Yamashiro

**Affiliations:** ^1^Sports Physiology Laboratory, Department of Health and Sports, Niigata University of Health and Welfare, Niigata, Japan; ^2^Graduate School of Health and Welfare, Niigata University of Health and Welfare, Niigata, Japan; ^3^Institute for Human Movement and Medical Sciences, Niigata University of Health and Welfare, Niigata, Japan; ^4^Japan Society for the Promotion of Science, Tokyo, Japan

**Keywords:** whole-hand water flow stimulation, primary somatosensory cortex (S1), paired-pulse inhibition (PPI), repetitive somatosensory stimulation, somatosensory evoked potentials (SEP)

## Abstract

Sustained peripheral somatosensory stimulations, such as high-frequency repetitive somatosensory stimulation (HF-RSS) and vibrated stimulation, are effective in altering the balance between excitation and inhibition in the somatosensory cortex (S1) and motor cortex (M1). A recent study reported that whole-hand water flow (WF) stimulation induced neural disinhibition in the M1. Based on previous results, we hypothesized that whole-hand WF stimulation would lead to neural disinhibition in the S1 because there is a strong neural connection between M1 and S1 and aimed to examine whether whole-hand WF stimulation would change the neural balance between excitation and inhibition in the S1. Nineteen healthy volunteers were studied by measuring excitation and inhibition in the S1 before and after each of the four 15-min interventions. The excitation and inhibition in the S1 were assessed using somatosensory evoked potentials (SEPs) and paired-pulse inhibition (PPI) induced by single- and paired-pulse stimulations, respectively. The four interventions were as follows: control, whole-hand water immersion, whole-hand WF, and HF-RSS. The results showed no significant changes in SEPs and PPI following any intervention. However, changes in PPI with an interstimulus interval (ISI) of 30 ms were significantly correlated with the baseline value before whole-hand WF. Thus, the present findings indicated that the whole-hand WF stimulation had a greater decreased neural inhibition in participants with higher neural inhibition in the S1 at baseline. Considering previous results on M1, the present results possibly show that S1 has lower plasticity than M1 and that the duration (15 min) of each intervention may not have been enough to alter the balance of excitation and inhibition in the S1.

## Introduction

Somatosensory function based on somatosensory information processing in the central nervous system plays a crucial role in our daily lives. Several brain regions are involved in somatosensory function, such as the primary somatosensory cortex (S1), posterior parietal cortex, presupplementary motor area, and basal ganglia (Pastor et al., 2004; Conte et al., 2012). In particular, several studies on healthy participants have reported that the delicate balance between excitatory and inhibitory processes in the S1 strongly affects somatosensory functions (Lenz et al., 2012; Rocchi et al., 2016) and is strongly related to synaptic changes (Abbott and Nelson, 2000; Carcea and Froemke, 2013), which are critically involved in motor skills, learning, and sensory functions (Dinse et al., 2003; Höffken et al., 2007).

Sustained peripheral somatosensory stimulation is effective for altering the balance between excitation and inhibition (Godde et al., 1996; Rocchi et al., 2017; Saito et al., 2018), such as high-frequency repetitive somatosensory stimulation (HF-RSS), in which a patterned electric stimulation is applied to the skin through surface electrodes, and tactile coactivation stimulation, which involved the application of simultaneous tactile stimuli at several sites of the hand. Both methodologies alter the balance between excitation and inhibition in the S1, with sustained stimulation resulting in an increase of the receptive field of the S1 and the number of neurons recruited for the response to sensory input (Godde et al., 1996, 2000; Dinse et al., 2003). Considering these mechanisms, sustained somatosensory stimulation to a larger site of the hand would change the balance between excitation and inhibition in the S1.

Recently, our research group developed whole-hand water flow (WF) stimulation in which sustained somatosensory stimulation by water flow to a large site of the hand is applied; this induces neural disinhibition in the primary motor cortex (M1) (Sato et al., 2015). *In vivo* experiments identified the anatomic substrate for this cross-systemic plasticity as topographically and functionally specific reciprocal connections between the primary motor cortex (MI) and the primary somatosensory cortex (SI) (Rocco and Brumberg, 2007). Other animal studies have shown that the M1 receives projections from homotopic and heterotopic S1 (Fabri and Burton, 1991; Izraeli and Porter, 1995). In humans, using diffusion MRI, a tractography study demonstrated that the M1 and S1 are directly connected through short U-shaped fibers running beneath the central sulcus (Catani et al., 2012). Additionally, recent neurophysiological mapping findings demonstrated that the sensory input to the S1 projected to homotopic and heterotopic M1 and that the sensorimotor integration involves center-inhibition and surround-facilitation in the M1 hand area (Dubbioso et al., 2017). Therefore, the previously observed plastic change in the M1 by whole-hand WF stimulation would be attributed to the change in the balance between excitation and inhibition in the S1 induced by the sustained somatosensory input. In addition, the whole-hand WF stimulation induces not only tactile but also proprioceptive stimulation from a large site of the hand (Sato et al., 2015), which may result in more neurons recruited for the response to sensory input and increase the size of the receptive field of the S1. That is, whole-hand WF stimulation may be a more powerful tool to observe plasticity induction in the S1.

The paired-pulse paradigm has become a standard procedure for evaluating the balance between excitation and inhibition in human participants. It involves the application of pairs of stimuli in close succession (paired-pulse stimulation), which can be used as a marker of intracortical excitability in the S1. In the case of the S1, paired-pulse inhibition (PPI) expresses the result of paired-pulse peripheral stimulations with a short interstimulus interval (ISI), in which the cortical responses to the second stimulus are more significantly suppressed compared to the first stimulus. The PPI is quantified by calculating the ratio of the amplitude of the second response divided by the amplitude of the first response. Small ratios indicate higher PPI, whereas large ratios indicate lower PPI, which is used as a marker for enhanced excitation.

The present study aimed to clarify the effect of whole-hand WF on the balance between excitation and inhibition in the S1 using a paired-pulse paradigm. As previous evidence has shown that whole-hand WF produces simultaneous somatosensory stimulation to a large area of the hand (Sato et al., 2015), we hypothesized that whole-hand WF would decrease PPI due to an increase in the receptive field of the S1 and the number of neurons recruited for the response to sensory input (Godde et al., 1996, 2000; Dinse et al., 2003). Elucidating the cortical somatosensory process induced by WF stimulation and its effects on the processing of other sensory inputs will help delineate the mechanisms of sensory integration and facilitate the development of improved aquatic therapies for patients with neurological disabilities.

## Materials and methods

### Participants

Nineteen healthy right-handed participants (10 men and 9 women; mean age, 23.10 ± 3.41 years; height, 165.13 ± 9.11 cm; weight 59.45 ± 11.10 kg) participated in the present study. None of the participants had a history of neurological or psychiatric diseases. All participants signed a written informed consent form, which provided a full explanation of this study and methods before the experimental session. This study was conducted in accordance with the Declaration of Helsinki and approved by the Ethics Committee of Niigata University of Health and Welfare (18712-210823).

### Interventions

**FIGURE 1:**
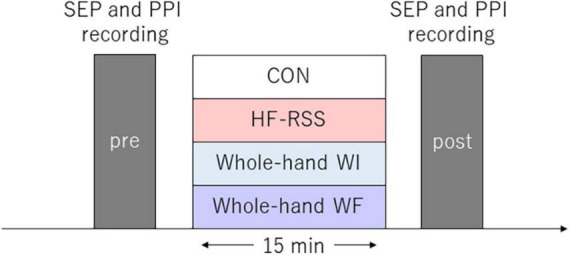
Experimental procedure. Somatosensory evoked potential and paired-pulse inhibition were assessed before and after each intervention for 15 min. Each intervention was conducted in random order for each participant.

The interventions (15 min) were as follows: non-immersed control (CON), whole-hand water immersion (WI), whole-hand WF stimulation (WF), and HF-RSS. In all interventions, the participants were instructed to place their right hand in the sluicing device, keeping it relaxed. The left hand was placed on a soft support beside the body and was kept relaxed. The hands were fixed in the same position for whole-hand WF interventions, using a belt to avoid muscle contractions. For whole-hand WF, WF was applied to the palm of the right hand using a sluicing device (Japan Aqua Tec, Sasebo, Nagasaki, Japan) at ∼40 L/min (Sato et al., 2015). In all the interventions, the participants were instructed to focus their gaze on the wall facing them throughout the experiments to divert their attention from their right hand. For each intervention by water, the ambient temperature was 29^°^C ± 1^°^C, and the water temperature was 33^°^C ± 1^°^C. The ambient and water temperatures were modulated to avoid changing the skin temperature. The experimental protocol is shown in Figure 1. All participants underwent these four conditions in random order, with an interval of at least 5 days between the experiments.

### High-frequency repetitive somatosensory stimulation

The HF-RSS consisted of 20-Hz trains of square wave electrical pulses of 200-μs duration delivered for 1 s, with 5-s intertrain intervals, for 15 min. Stimuli were administered using a constant current stimulator (Electronic Stimulator SEN-7023; Nihon Kohden Co., Tokyo, Japan) through the isolator (SS-104; Nihon Kohden Co., Tokyo Japan) to surface adhesive electrodes of an approximately 1 cm^2^ area, with the anode located on the distal phalanx of the right index finger and the cathode located on the proximal phalanx of the same finger. The intensity was set to the maximal intensity tolerated without pain minus 0.1 mA (Ragert et al., 2008; Kattenstroth et al., 2012; Schlieper and Dinse, 2012; Rocchi et al., 2017).

### Somatosensory evoked potentials and paired-pulse inhibition recording and analysis

Somatosensory input from peripheral nerves activates several cortical areas, and this modulation can be evaluated using somatosensory evoked potentials (SEPs). We measured SEPs before and after each intervention, according to a previously described methodology (Lenz et al., 2012; Rocchi et al., 2016; Saito et al., 2018). SEPs were recorded, and the N20-P25 component from the active electrode was placed at C3’ (located 2 cm posterior to C3), and the reference electrode was placed at Fz, according to the international 10–20 system using a Brain Products amplifier system (Brain Products GmbH, Gilching, Germany) and Brain Vision Professional Recorder (Brain Products GmbH, Gilching, Germany).

Single-pulse SEP and PPI were measured to assess the balance between cortical excitation and inhibition in the S1. Paired-pulse electrical stimulation of the median nerve was applied with ISIs of 5 ms and 30 ms in combination with SEP recording. The ISIs were determined in two separate sequences based on the methods reported in previous studies (Höffken et al., 2007; Ragert et al., 2008; Rocchi et al., 2016, 2017). PPIs have different GABAergic modulations depending on the ISI. PPIs with ISI of 5 and 30 ms were mainly modulated by synaptic GABAA and GABAB receptor activities, respectively (Chowdhury and Rasmusson, 2003). Based on this observation, the present study measured PPIs with two ISI of 5 and 30 ms to examine the mechanism of WF stimulation-induced plasticity in S1. Single-pulse and paired-pulse stimulations were randomly applied at a frequency of 2 Hz, which was controlled by a pulse control system (Pulse Time II; Medical Try System, Tokyo, Japan). The participants were seated in a comfortable chair and instructed to relax but stay awake with their eyes closed. Median nerve stimulation was performed with a surface electrode placed on the right wrist with constant current stimulation (Electronic Stimulator SEN-7023; Nihon Kohden Co., Tokyo Japan) through an isolator (SS-104; Nihon Kohden Co., Tokyo, Japan) with the anode placed on the wrist crease and the cathode placed 2 cm proximal. The two electrodes were attached with double-faced tape and covered with a waterproof transparent film (Tegaderm Hydrocolloid Dressing; 3M Japan, Tokyo, Japan). A monophasic wave pulse of 200-μs duration was delivered at 250% of the sensory threshold and a frequency of 2 Hz. For each type of stimulus, 400 stimulation trials were performed, and the sequences were randomized (1,200 stimulations). All the data were collected at a sampling rate of 5 kHz. Continuous electroencephalogram data were band-pass filtered at 3–2 kHz, and the signal was recorded from –20 to 100 ms with regard to the pulse. The 20-ms period before the stimulus was the baseline. Epochs with responses exceeding ± 70 μV were rejected from the analysis, and the remaining data were averaged.

**FIGURE 2:**
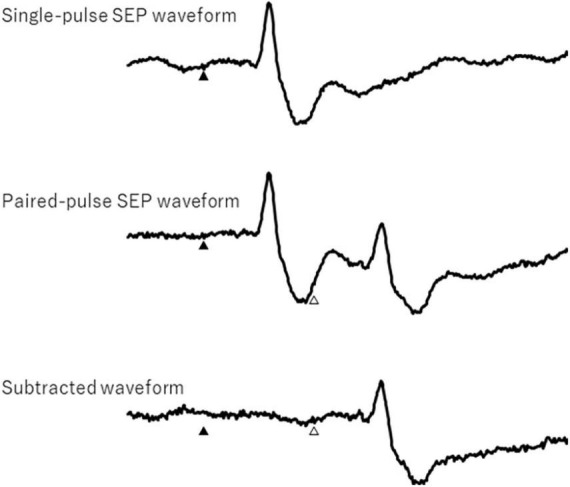
Single- and paired-pulse somatosensory evoked potential waveforms and subtracted waveform. Filled and open triangles show first and second electrical stimulation, respectively.

The peak-to-peak amplitudes of the cortical N20 and P25 response components for the first and second paired-pulse stimulations were analyzed. In the paired-pulse trial, the responses after the second response were obtained by subtracting a single SEP (Figure 2). Each PPI was calculated as the ratio of the response of a single pulse and the second response of the subtracted paired pulse.

### Statistical analyses

Single-pulse SEP amplitudes (N20, P25, and N20-P25), latency (N20, P25), PPI ratio (N20, P25, N20-P25), and sensory thresholds were analyzed using statistical software (SPSS version 18; IBM, Chicago, USA).

The distributions of all data were confirmed by the Shapiro–Wilk test. Sensory threshold was entered into a Friedman test with “intervention” (CON, HF-RSS, WI, and WF) as the within-subject factor. Other parameters were entered into a generalized linear mixed model (GLMM) with “intervention” and “time” (pre and post) as the within-subject factors.

Pearson or Spearman correlation analysis was performed to assess the relationship between PPI at pre-intervention and the change in PPI with each ISI. Statistical significance was set at *p* < 0.05. All data are expressed as the mean ± standard deviation.

## Results

**FIGURE 3:**
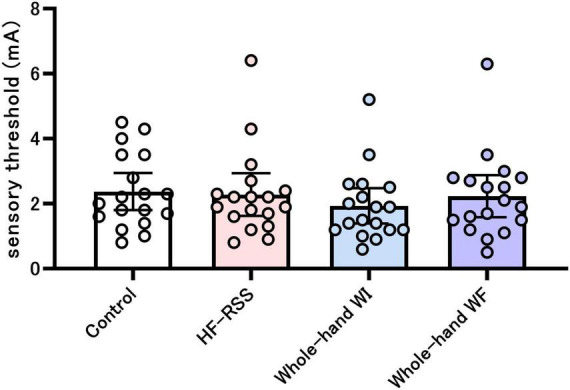
Sensory threshold before each intervention. There was no significant difference among the four interventions, as shown by the Friedman test. HF-RSS, high-frequency repetitive somatosensory stimulation.

Figure 3 shows the sensory thresholds before each intervention. The sensory threshold did not change significantly across the four interventions (X^2^ = 5.654, *p* = 0.13).

**FIGURE 4:**
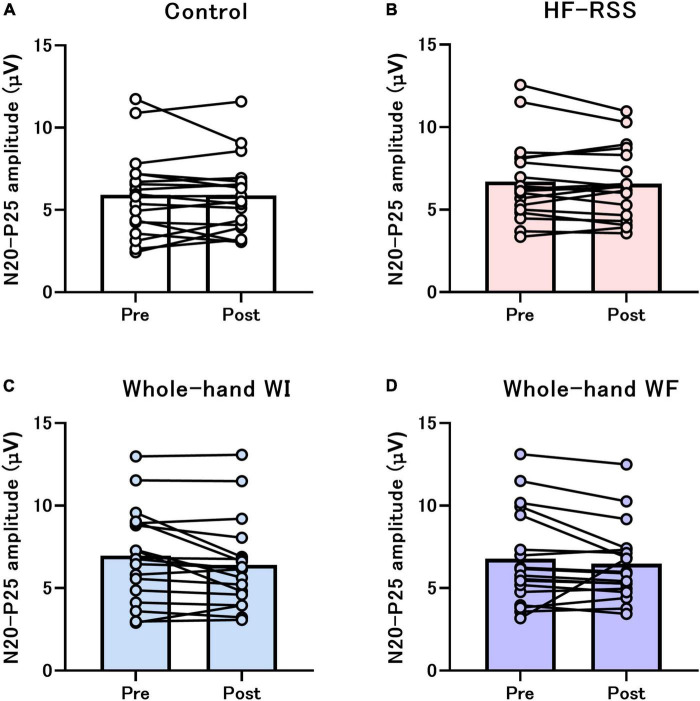
Single-pulse N20-P25 amplitude before and after each intervention: **(A)** Control, **(B)** HF-RSS, **(C)** Whole-hand WI, and **(D)** Whole-hand WF. Generalized linear mixed model revealed that there was no significant interaction or main effect of “intervention” and “time,” except for the main effect of “intervention.” HF-RSS, high-frequency repetitive somatosensory stimulation.

For single-pulse SEP, GLMM revealed no significant interactions and main effect in N20, P25, and N20-P25 peak-to-peak, except for the main effect of “intervention” in N20-P25 peak-to-peak (Table 1 and Figure 4).

**TABLE 1:** Statistical results of single-pulse SEP components.

		df	*F*-value	*P*-value
N20 amp.	Intervention	(3,126)	2.383	0.073
	Time	(1, 126)	1.549	0.216
	Interaction	(3, 126)	0.460	0.710
N20 lat.	Intervention	(3,126)	1.771	0.156
	Time	(1, 126)	1.546	0.216
	Interaction	(3, 126)	2.137	0.099
P25 amp.	Intervention	(3,126)	2.003	0.117
	Time	(1, 126)	1.455	0.230
	Interaction	(3, 126)	1.864	0.139
P25 lat.	Intervention	(3,126)	1.144	0.334
	Time	(1, 126)	0.336	0.563
	Interaction	(3, 126)	0.397	0.755
N20-P25	Intervention	(3,126)	4.112	0.008
	Time	(1, 126)	3.139	0.079
	Interaction	(3, 126)	0.610	0.610

Amp., amplitude; lat., latency; N20-P25, N20-P25 peak-to-peak; SEP, somatosensory evoked potential.

**FIGURE 5:**
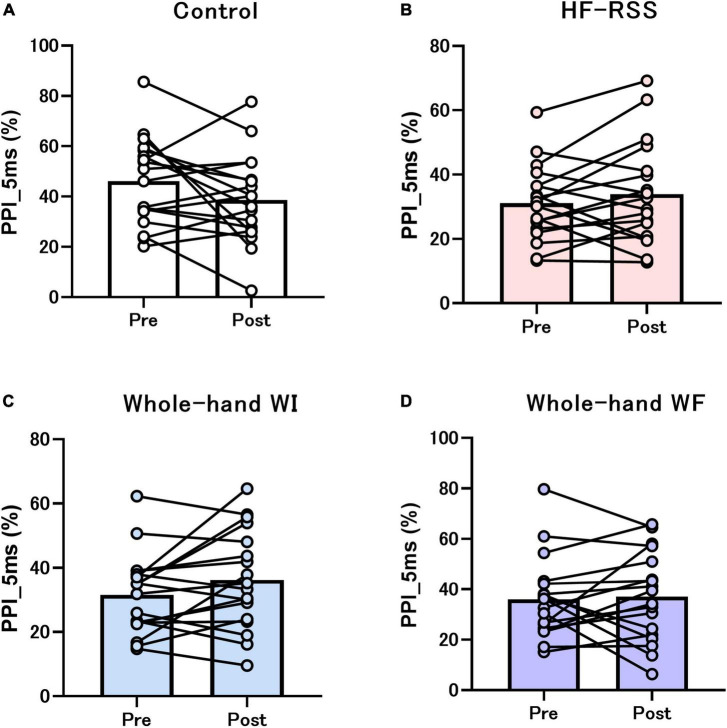
PPI_5 ms of N20-P25 amplitude before and after each intervention: **(A)** Control, **(B)** HF-RSS, **(C)** Whole-hand WI, and **(D)** Whole-hand WF. Generalized linear mixed model revealed that there was no significant interaction or main effect of “intervention” and “time.” HF-RSS, high-frequency repetitive somatosensory stimulation.

**FIGURE 6:**
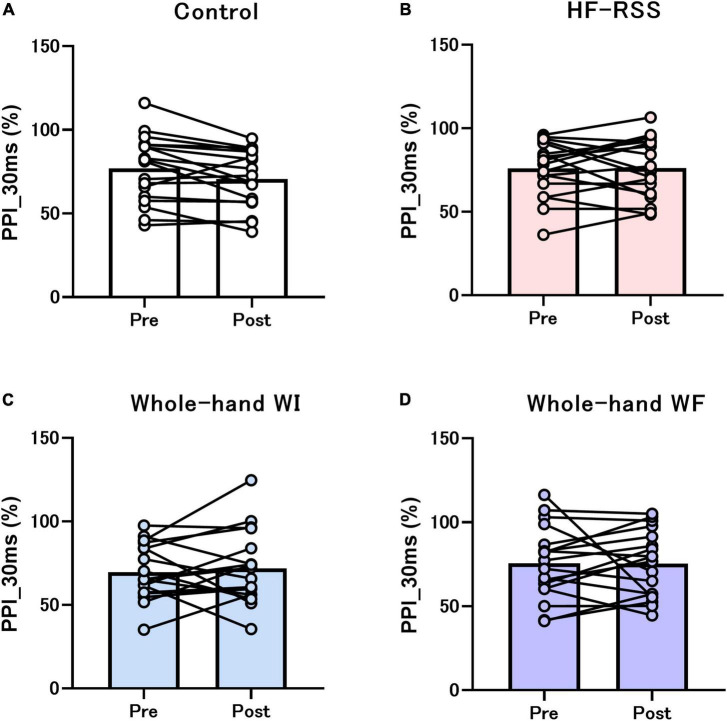
PPI_30 ms of N20-P25 amplitude before and after each intervention: **(A)** Control, **(B)** HF-RSS, **(C)** Whole-hand WI, and **(D)** Whole-hand WF. Generalized linear mixed model revealed that there was no significant interaction or main effect of “intervention” and “time.” HF-RSS, high-frequency repetitive somatosensory stimulation.

Figures 5, 6 present the PPI_5 ms and PPI_30 ms before and after each intervention, respectively. GLMM revealed no significant interactions [PPI_5 ms: *F* (3, 51) = 2.319, *p* = 0.086, η^2^*p* = 0.120; PPI_30 ms: *F* (3, 51) = 0.792, *p* = 0.504, η^2^*p* = 0.045]. There was no main effect of “intervention” [PPI_5 ms: *F* (2.116, 35.965) = 3.080, *p* = 0.056, η^2^*p* = 0.153; PPI_30 ms: *F* (1.863, 31.674) = 0.218, *p* = 0.790, η^2^*p* = 0.013] and “time” [PPI_5 ms: *F* (1, 17) = 0.071, *p* = 0.792, η^2^*p* = 0.004; PPI_30 ms: *F* (3, 17) = 0.239, *p* = 0.631, η^2^*p* = 0.014].

**FIGURE 7:**
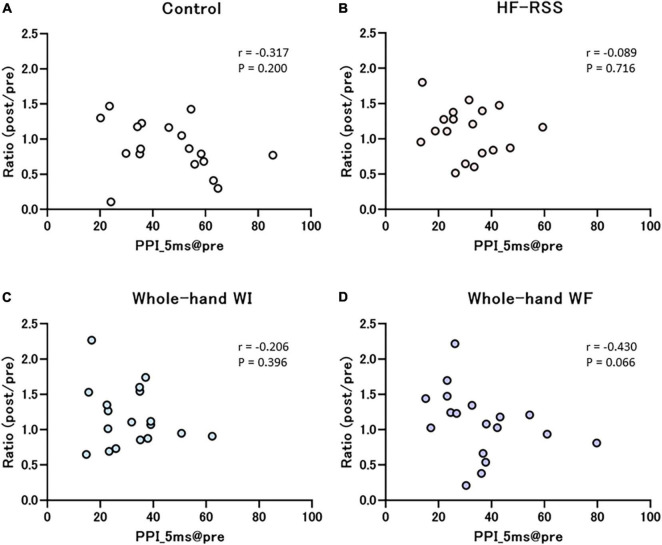
Correlation between PPI_5 ms at pre-intervention and the ratio of before to after each intervention: **(A)** Control, **(B)** HF-RSS, **(C)** Whole-hand WI, and **(D)** Whole-hand WF. There was no significant correlation in any intervention. HF-RSS, high-frequency repetitive somatosensory stimulation.

**FIGURE 8:**
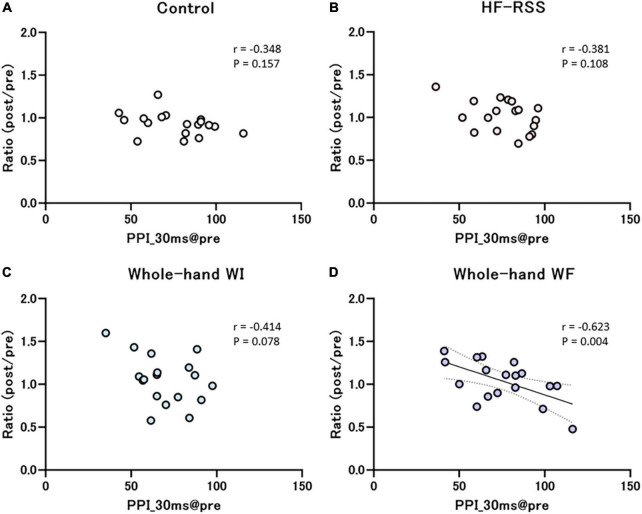
Correlation between PPI_30 ms at pre-intervention and the ratio of before to after each intervention: **(A)** Control, **(B)** HF-RSS, **(C)** Whole-hand WI, and **(D)** Whole-hand WF. There was a significant negative correlation only in whole-hand water immersion, but not in other interventions. HF-RSS, high-frequency repetitive somatosensory stimulation.

Figures 7, 8 present the relationship between each PPI at pre-intervention and the ratio of PPI with ISIs in HF, WI, and WF interventions. The PPI at pre-intervention was significantly correlated with the ratio of PPI_30 ms in WF alone (*r* = –0.589, *p* = 0.010).

## Discussion

The present data showed that 15-min whole-hand WF, HF-RSS, whole-hand WI, and CON did not affect the balance between excitation and inhibition in the S1, as measured by the N20-P25 amplitude of SEP and PPI. Additional findings revealed that the change in PPI with longer ISI (30 ms) was negatively correlated with the baseline value in whole-hand WF.

The PPI reflects the inhibitory influence of the first stimulus on the response to the second stimulus. This technique is useful for investigating changes in the balance between cortical excitation and intracortical inhibition. The present results indicate that 15-min whole-hand WF and other interventions do not change the balance of neural excitation and inhibition at both cortical and subcortical levels of the somatosensory afferent pathway. Animal studies have suggested that, following the previous activation of pyramidal cells, calbindin (CB)-expressing inhibitory interneurons inhibit pyramidal cells within the microcolumn through synapses on superficial dendrites (Gulyás and Freund, 1996). Additionally, pyramidal cells synapse on parvalbumin (PV)-expressing inhibitory interneurons *via* N-methyl-D-aspartate receptors located within layers IV/V of SI (Labedi et al., 2014). These PV cells synapse perisomatically on the pyramidal cell itself and synapse with the basal dendrites of pyramidal cells in neighboring macrocolumns (Blatow et al., 2003; Freund, 2003; Markram et al., 2004; Howard et al., 2005; Feldmeyer et al., 2018). The inhibition provided by CB-expressing, perisomatic PV-expressing, and lateral PV-expressing cells may account for the overall inhibition of pyramidal cells and thus contribute to the reduced amplitude of SEP induced by the second stimulus compared to the first one. The N20 and P25 components of the SEP are generated in the posterior bank of the central sulcus and anterior crown of the postcentral gyrus, respectively (Allison et al., 1989; McCarthy et al., 1991). PPI at a shorter ISI of 5 ms is generally hypothesized to be due to inhibitory interactions in the S1 (Emori et al., 1991; Ugawa et al., 1996). At longer ISIs, inhibition of the N20-P25 component may involve subcortical structures within the somatosensory pathway, such as the dorsal column nuclei or thalamus (Lüders et al., 1984; Höffken et al., 2010). Therefore, no change in PPI with all ISI suggests that the present interventions, including whole-hand WF, whole-hand WI, and HF-RSS in 15 min, do not affect inhibitory interactions at the cortical (S1) and subcortical levels.

Paired-pulse evoked inhibition has been reported to occur regardless of modality in the motor, visual, auditory, and somatosensory cortices (Kujirai et al., 1993; Carandini et al., 2002; Freeman et al., 2002; Shapley et al., 2003; Fitch et al., 2008; Lenz et al., 2012; Gómez-Nieto et al., 2020). In the motor domain, paired-pulse transcranial magnetic stimulation has been widely used to study the intracortical inhibition of the human motor cortex. Previous pharmacological studies have provided several lines of evidence for the critical role of GABAergic, presumably GABAA-modulated, inhibition (Kujirai et al., 1993; Ziemann et al., 1996), although the involvement of GABAB has also been advocated (Porter and Nieves, 2004). Our previous study examined paired-pulse modulated inhibitory circuits after whole-hand WI and WF and showed that whole-hand WF induces temporary attenuation of GABAA-modulated inhibition in the M1, but not WI (Sato et al., 2015). Considering these results in the M1, we hypothesized that whole-hand WF would decrease PPI in the S1, but not in line with the present hypothesis.

A possible explanation for this is the difference in neural plasticity between S1 and M1. A previous study demonstrated that quadripulse transcranial magnetic stimulation (QPS), which is the most powerful and reliable non-invasive brain stimulation method to induce neural plasticity in humans (Matsumoto and Ugawa, 2020), was applied to S1 and M1 separately and significantly increase the neural excitability in M1 but not S1 (Nakatani-Enomoto et al., 2012). This may indicate that M1 has higher plasticity than S1. Therefore, no plasticity change in S1 after all interventions in the present study would be due to the lower plasticity of the S1 compared to M1. Another possible explanation for this is the shorter duration of intervention (15 min) to induce plastic alterations in the S1. Almost all previous studies reported that 30 min or more of peripheral electrical stimulation affects the balance between excitatory and inhibitory circuits (Freyer et al., 2013; Rocchi et al., 2017; Saito et al., 2018). However, the response to peripheral electrical stimulation is inconsistent across stimulus durations, such as increased PPI_5 ms after a 45-min intervention (Rocchi et al., 2017), decreased PPI_30 ms after a 3-h intervention (Höffken et al., 2007), and unchanged PPI_100 ms after a 30-min intervention (Saito et al., 2018). Additionally, considering the present results in line with the hypothesis that the present HF-RSS would not change S1 excitability due to its short stimulus duration, it is possible that a stimulation time of 45 min or longer is required for peripheral stimulation, including whole-hand WF, whole-hand WI, or HF-RSS, to alter S1 excitability.

Interestingly, we found that the higher the PPI_30 ms at pre-intervention, the more the disinhibition after whole-hand WF intervention, but not after other interventions. The results, in which the participants with higher PPI_30 ms at pre-intervention had more disinhibition after whole-hand WF, could be explained by the relationship between the cortical inhibition and neural plasticity. Previous studies have reported that lower cortical inhibition, when assessed by a paired-pulse paradigm, was related to lower neural plasticity in S1 (Erro et al., 2018). Cortical inhibitory circuits finely tune and regulate the neural interaction and induce the plastic change in the cerebral cortex [see review by Artinian and Lacaille (2018)]. Therefore, more disinhibition by whole-hand WF intervention could be attributed to higher inhibitory circuit in S1. In addition, we wondered why this correlation was observed only during the whole-hand WF intervention. This result may be due to the distinct somatosensory inputs induced by WF and other factors. Whole-hand WF has been reported to induce tactile and vibratory stimulations by skin movement, whereas tactile stimulation alone has been reported in whole-hand WI (Sato et al., 2015) and HF-RSS (Rocchi et al., 2017). Because somatosensory inputs involving skin and muscle spindle movements reach BA3a, 2, and 4, whereas tactile inputs mainly reach BA3b and 1, whole-hand WF and other interventions may affect different inhibitory circuits (Cooper et al., 1975; Jones, 1983; Terumitsu et al., 2009). Regarding the relationship between inhibitory circuits and PPI at different ISIs, pre- and post-synaptic GABAB receptor antagonists induced more inhibition at ISIs longer than 25 ms, whereas post-synaptic GABAA receptor antagonists induced more inhibition at ISIs of 5 ms than at longer ISIs, although both antagonists produced disinhibition at ISIs of 5 ms and more (Chowdhury and Rasmusson, 2003). Further pharmacological studies are required to confirm this possibility.

The sensorimotor cortex hyperexcitability represents an important pathogenic mechanism underlying several neurological diseases such as familial adult myoclonic epilepsy type 2 (FAME2) (Dubbioso et al., 2022), ALS (Ranieri et al., 2020), dystonia (Erro et al., 2018), fibromyalgia (Lim et al., 2015), and schizophrenia (Daskalakis et al., 2020). These diseases are typically caused by the abnormal neural balance between excitation and inhibition. Therefore, the present result in which the change in PPI with longer ISI (30 ms) was negatively correlated with the baseline value in whole-hand WF suggests the potential effect of whole-hand WF on hyperexcitability in neurological diseases. However, since the present results were obtained from healthy young participants, future studies are needed with such patients.

The present study has several limitations. First, the sample size is small; it was determined on the basis of previous studies, whereas it should have been based on a power analysis. Second, we measured only SEP and PPI to evaluate S1 excitability. To clarify the change in neural balance between excitation and inhibition in the S1 in greater detail, it would be preferable to investigate other biomarkers of inhibitory function such as early and late components of SEP high-frequency oscillation (e- and l-HFO) (Erro et al., 2018; Dubbioso et al., 2022; Tomasevic et al., 2022), which reflect the activity of thalamocortical fibers directed to areas 3b and 1 within S1 and intracortical inhibition in S1, putatively a result of local GABAergic interneurons. Third, the effects of the present interventions on several somatosensory functions remain unclear. Previous studies indicate that PPIs are related to some behavioral parameters such as somatosensory temporal discrimination threshold (STDT) and two-point discrimination (TD) performance. Interestingly, STDT and TD performance have been reported to be modulated by PPI with a shorter ISI of 5 ms (Tamura et al., 2008; Rocchi et al., 2016) and longer ISI of 30 ms (Lenz et al., 2012), respectively. Therefore, future research should examine whether the change in PPI by whole-hand WF modulates behavioral performance. Finally, we did not examine whether whole-hand WF affects GABAergic neural activities because we used indirect non-invasive evaluation for GABAergic intracortical inhibition in S1 in the present study.

## Conclusion

The present data showed that 15-min whole-hand WF, HF-RSS, whole-hand WI, and CON did not affect the balance between excitation and inhibition in the S1, as measured by the N20-P25 amplitude of SEP and PPI. Additional findings revealed that the change in PPI with longer ISI (30 ms) was negatively correlated with the baseline value in whole-hand WF. These results indicated that even a 15-min whole-hand WF can induce intracortical disinhibition in the participants with high inhibition in S1, which raises the possibility that whole-hand WF could be a useful tool for improving somatosensory function.

## Data availability statement

The raw data supporting the conclusions of this article will be made available by the authors, without undue reservation.

## Ethics statement

The studies involving human participants were reviewed and approved by the Ethics Committee of Niigata University of Health and Welfare. The patients/participants provided their written informed consent to participate in this study.

## Author contributions

DS and KY contributed to conception and design of the study. DC, KI, and DS performed the experiment and statistical analysis. DC and DS wrote the first draft of the manuscript. TF and GO wrote sections of the manuscript. All authors contributed to manuscript revision, read, and approved the submitted version.
